# Predictors of breast cancer screening among women of reproductive age in Tanzania: Evidence from DHS 2022

**DOI:** 10.1371/journal.pone.0298996

**Published:** 2024-11-01

**Authors:** Jovin R. Tibenderana, Sanun Ally Kessy, Dosanto Felix Mlaponi, Ndinagwe Lloyd Mwaitete, John Elyas Mtenga

**Affiliations:** 1 Department of Epidemiology and Biostatistics, Kilimanjaro Christian Medical University College, Moshi, Tanzania; 2 Institute of Public Health, Kilimanjaro Christian Medical University College, Moshi, Tanzania; University of Environment and Sustainable Development, GHANA

## Abstract

**Background:**

Breast cancer is a global concern, with 2.3 million new cases and 685,000 deaths recorded in 2020, and projections of reaching 4.4 million cases by 2070. In Tanzania, it’s the second leading cause of cancer-related deaths among women, often diagnosed at advanced stages, leading to poor outcomes. Only 5% of women in the country report undergoing breast cancer screening, the aim study is to determine factors associated with breast cancer screening in Tanzania.

**Methods:**

This was analytical cross-sectional study among women of reproductive age in Tanzania, utilizing data from the Demographic and Health Surveys (DHS) which employed a two-stage probability sampling. A weighted sample of 15,189 women of reproductive age (15–49) was included in the study. Binary logistic regression analysis was used to examine factors associated with breast cancer screening. These results were presented using adjusted odds ratio (AOR) with a 95% confidence interval.

**Results:**

After controlling for other factors, the following factors remained significantly associated with breast cancer screening among women of reproductive age; age(AOR = 5.33, 95% CI 3.72, 7.63), being wealthy (AOR = 2.34, 95% CI 1.61, 3.38), residing in rural(AOR = 0.59, 95% CI 0.46, 0.763), being educated(AOR = 2.43, 95% CI 1.60, 3.68), being insured(AOR = 2.40, 95% CI 1.89, 3.06), healthcare facility visits in the past 12 months(AOR = 1.43, 95% CI 1.14, 1.78) and living in Northern zone (AOR = 2.43, 95% CI 1.42, 4.15) compared to western zone.

**Conclusion:**

Breast cancer screening is still under-utilized and have shown to be marginalized in women of reproductive age. Upgrading diagnostic services, comprehensive health education and awareness campaigns are instrumental to increase utilization and reduction of burden of breast cancers in Tanzania.

## Introduction

In the year 2020, there were around 2.3 million newly diagnosed cases of breast cancer and 685,000 deaths were attributed to breast cancer globally [1, 2] and cases are expected to rise up to 4.4 million in 2070 [3]. Among women, breast cancer accounted for approximately 24.5% of all cancer cases and 15.5% of cancer-related deaths, making it the leading cause of both incidence and mortality in most countries worldwide in 2020. [2].

Low- and middle-income countries (LMICs) and developing countries have experienced a significant rise in both the incidence and mortality rates of breast cancer. These countries also have much lower 5-year survival rates for breast cancer at around 53% [2, 4, 5]. In Africa, the primary focus has traditionally been on communicable diseases like tuberculosis, resulting in less attention to non-communicable diseases (NCDs) such as cancers. Consequently, this has led to very low rates of breast cancer screening. [5].

After cervical cancer, breast cancer stands as the second most prevalent cancer and the second leading cause of cancer-related deaths among women in Tanzania [6]. Tanzanian women face a lifetime risk of 1 in 203 of developing breast cancer, and more than 50% of those diagnosed will succumb to the disease and its related complications [7]. In Tanzania, 80% of all women diagnosed with breast cancer are identified at late at stages, specifically stages III and IV, where outcomes are poor and treatment is less effective [6–8]. Notably awareness remains a significant issue. Morse and colleagues reported that 44% of women had never heard of self-breast exams, and 32% were unaware of clinical breast examinations [9].

In Tanzania, 14.4% of newly diagnosed malignancies in women are breast cancers. The number of newly diagnosed cases of breast cancer in Tanzania is expected to rise by 82% by 2030 [6]. Tanzania has an age-standardized incidence rate of 19.4/100,000 women with breast cancer and an age-standardized death rate of 9.7/100,000. This results in a mortality-to-incidence ratio (MIR) of 0.5 [6, 10, 11]. In 2022, breast cancer accounted for 15.9% of all newly diagnosed cancers among females in Tanzania, making it the second most common newly diagnosed cancer for both males and females [10]. The Tanzania national guideline seeks to ensure early diagnosis and treatment of breast cancer in symptomatic, average-risk patients to improve quality of life and survival rates, while also recommending against a nationwide screening program until essential services are established, supporting opportunistic screening where feasible [12].

Tanzania Demographic health survey 2022 reported that only 5% of women aged 15–49 had been screened for breast cancer [13]. Most of the studies in Tanzania have studied factors associated with awareness of breast cancer screening [6, 9, 14].

Screening can effectively reduce mortality, morbidity as well as poor quality of life from breast cancer [15, 16]. For early detection of breast cancer, methods such as self-breast examination, clinical breast examination, and mammography should be employed, these methods are reported to reduce the rate of mortality from breast cancer by 25–30% [17].

A multi-country study has revealed significant associations between breast cancer screening and various factors. These include higher educational attainment, advanced age, possession of health insurance, elevated socio-economic status, and ownership of a television [5]. The true extent of breast cancer prevalence is not accurately represented in many Sub-Saharan African (SSA) countries, as most of the studies have not reported national burdens, leading to underreporting and a lack of a genuine reflection of the disease burden [18].

Research on breast cancer screening in Tanzania is limited and little is known about factors associated with breast cancer screening in the country [19]. This baseline knowledge is crucial for informing educational programs aimed at enhancing understanding and focusing on evidence-based, lifestyle-oriented interventions for breast cancer screening. Additionally, it is vital for promoting early detection and treatment of breast cancer. It’s crucial therefore to identify factors affecting breast cancer screening, hence this study objective is to determine factors associated with breast cancer screening in Tanzania.

## Material and methods

### The data source

The study utilized the Tanzania Demographic and Health Survey (TDHS) data, a nationwide cross-sectional survey conducted every 5 years [20]. TDHS 2022 is the most recent data which collected data on breast cancer screening among women of reproductive age. Tanzania is the largest country in East Africa, covering 940,000 square kilometers, 60,000 of which are inland water. The population of Tanzania as of 2022 was estimated to be 61,741,120 with an annual population growth rate of 3.2% [20, 21]. Tanzania’s healthcare infrastructure, regional disparities and barriers such as limited availability of screening services, emphasis on preventive rather than curative care, and limitations of health insurance impact breast cancer screening rates [22].

This was an analytical cross-sectional study conducted using nationally representative secondary data from the Tanzania demographic and health surveys (TDHS) of 2022. Women’s data was explored. DHS is national representative data which is funded by the U.S Agency for International Development and implemented by the Ministry of Health (MoH) (Tanzania Mainland), Ministry of Health (MoH) (Zanzibar), National Bureau of Statistics (NBS), Office of the Chief Government Statistician (OCGS) and technical support from ICF international [20–23]. The survey was conducted using face-to-face questionnaire interviews and used stratified design based on geographical areas (urban and rural), multistage cluster sampling to collect information about population health status, neonatal mortality, health behaviors, nutritional status family planning and demographics.

First, clusters (629) were identified and households were then selected randomly and on the distribution Probability Proportion to Size (PPS) was used. Among these, 26 households were systematically chosen as representative from each cluster comprising a total of 16,354 households. Eligibility for inclusion was based on all women 15–49 years old present in the sampled household the night before the interview, as this age group represents the reproductive years, during which healthcare access, awareness, and behaviors may influence breast cancer screening. Additionally, the presence of the participants the night before ensures accurate data collection from those residing in the area Women who were in hospital, prison, hotels, barracks and camps were excluded. Detailed information on sampling procedure and design has been previously reported [13].

### Variables

The dependent variable for this study was breast cancer screening, which was measured by a question ‘Has a doctor or other healthcare provider examined your breasts to check for breast cancer?’ and the detailed information on the breast cancer screening has been published elsewhere [19]. The binary response was Yes/No, following the approach employed by other researchers who utilized DHS data [5, 24]. Women who were unaware of their screening status were not included in this study.

The independent variables were social-demographic and socio economic factors, furthermore the association between breast cancer screening and various factors including age, wealth index, residence, number of living children, marital status, education, health insurance, employment, pregnancy status, house hold ownership of radio or television, healthcare facility visits in the past 12 months, breast feeding status and geographical zones were investigated as reported by other researchers [5, 23, 24]. Recategorization of wealth index from five to three categories combining poorest and poorer as ‘poor’ middle wealth as ‘middle’ and richer and richest as ‘rich’ was done aligning with previous research practices [5, 25, 26].

Additionally, the age of survey respondents was recorded as continuous variable, which was grouped into three categories 15–24, 25–34 and 35–49 years old as others researchers [5, 25]. Furthermore, mothers employment status was recategorized into two categories ‘working’ and ‘not working’ as previously categorized [27, 28].

### Data management and analysis

Data cleaning and analysis were conducted using Stata 18. Categorical variables were summarized using frequencies and percentages to provide clear and concise insights into the distribution of responses within each category for further analysis. The Pearson Chi-squared test investigated the association between breast cancer screening and participants characteristics. The sample was weighted (v005/1,000,000) to address any over- and under-sampling issues, and the survey set(svy)command in Stata was used in the analysis to account for the survey’s complex design. Logistic regression was employed was carried out to assess associations between dependent and independent variables with 95% confidence intervals (CI). The variables associated at binary logistic regression with a significance level (p ≤ 0.20) [17], were entered into multiple logistic regression to identify key determinants while controlling for potential confounding effects. Statistical significance was indicated at a p-value of 0.05, and predictors of the outcome variable were identified accordingly. The variables reported were those found to be significantly associated based on adjusted odds ratios because they indicate a stronger, more reliable association with the outcome and make it easier for readers to focus on key insights.

### Ethical consideration

The formal written request was submitted to the DHS program and approval was given to access and utilize data from http://www.dhsprogram.com. The questionnaire for standard DHS was reviewed and approved Medical Research Council of Tanzania and the Zanzibar Health Research Institute and ICF’s Internal Review Board (IRB). Participants provided either written or verbal informed consent before participating in the survey. Respondents were not subjected to any form of coercion and all data are protected ensuring no any personally identifiable information [5, 28, 29].Further details on ethical consideration are available elsewhere [30].

## Results

### Participant characteristics

Among 15,188 participants of this study, the mean (SD) age was 29.3(9.8) with few (30.2%) of the respondents were aged between 25–34, where more than half (53.2%) had primary education. More than half of women (60.7%) were either married or living with a partner. The majority (64.3%) of women were from rural while some women in this study were not working (35.7%). Slightly less than half of the study participants were from rich households (48.1%) and almost all (94.2%) of the study participants were un insured. Around half (53.9%) of women did not have radios in their households while some (32.6%) had television. More than half (53.0%) of the participants had history of visiting healthcare facility for the past 12 months prior the survey. Of all participants 92.3% were not pregnant nor did they know their pregnancy status. Around half of the participants (55.7%) had 1–4 living children. Few of the study participants were breastfeeding during the survey (22.9%) and very few (2.6%) of respondents were from Western zone (Table 1).

**Table 1:** Socio-demographic characteristics of the study participants (weighted N = 15, 189).

Variable	Frequency (n)	Percentage (%)
**Age group(years)**		
15–24	5,778	38.0
25–34	4,591	30.2
35–49	4,821	31.7
**Education**		
No education	2,430	16.0
Primary	8,087	53.2
Secondary/higher	4,673	30.8
**Marital status**		
Never married	4,022	26.5
Married/living with partner	9,220	60.7
Widowed/divorced/separated	1,948	12.8
**Residence**		
Urban	5,428	35.7
Rural	9,761	64.3
**Employment status**		
Not working	5,424	35.7
Working	9,765	64.3
**Wealth Index status**		
Poor	5,015	33.0
Middle	2,870	18.9
Rich	7,304	48.1
**Health insurance**		
No	14,302	94.2
Yes	887	5.8
**Household has radio**		
No	8,191	53.9
Yes	6,998	46.1
**Household has television**		
No	10,240	67.4
Yes	4,950	32.6
**Visited healthcare facility last 12 months**		
No	7,134	47.0
Yes	8,055	53.0
**Pregnancy status**		
No or unsure	14,013	92.3
Yes	1,177	7.7
**Number of living children**		
None	3,950	26.0
1–4	8,457	55.7
>4	2,783	18.3
**Breastfeeding status**		
No	11,713	77.1
Yes	3,476	22.9
**Zones**		
Western zone	1,266	8.3
Northern zone	1,731	11.4
Central zone	1,569	10.3
Southern highlands	924	6.1
Southern zone	803	5.3
Southwest highlands	1,322	8.7
Lake zone	4,406	29.0
Eastern zone	2,651	17.5
Zanzibar	516	3.4

### Self-reported breast cancer screening by socio demographic characteristics

Table 2 describes proportions on self-reported breast cancer, the overall proportion of breast cancer among women of reproductive age in Tanzania in 2022 was found to be 5.2%. Specifically, the highest proportion of breast cancer screening was found among those aged 35–49 (9.2%), respondents who had secondary education (6.9%), those participants whose marital status was either widowed, divorced or separated (7.7%), urban residents (8.7%), respondents who were working (6.3%). Similarly, the proportion of breast cancer screening was higher among participants from rich households (8.0%), those who were insured (17%), those whose household had radio (6.6%) and television (8.4%), participants who visited health facility for past 12 months (6.6%), those who had no or were unsure of they were pregnant (5.3%). Additionally, the proportion was higher among women who had 1–4 living children (6.6%), those who were not breastfeeding (5.4%) and women from eastern zone (7.3%)

**Table 2:** Weighted: Self-reported breast cancer screening by participant characteristics (N = 15,189).

Variable	Ever screened for breast cancer					Chi-square (P-value)
	No			Yes		
	n	%		n	%	
**Age group(years)**						<0.001
15–24	5,674	98.2		103	1.8	
25–34	4,350	94.8		241	5.2	
35–49	4,376	90.8		445	9.2	
**Education**						<0.001
No education	2,380	98.0		49	2.0	
Primary	7,669	94.8		417	5.2	
Secondary/higher	4,351	93.1		322	6.9	
**Marital status**						<0.001
Never married	3,910	97.2		112	2.8	
Married/living with partner	8,692	94.3		528	5.7	
Widowed/divorced/separated	1,799	92.3		149	7.7	
**Residence**						<0.001
Urban	4,953	91.3		475	8.7	
Rural	9,447	96.8		314	3.2	
**Employment status**						<0.001
Not working	5,248	96.8		176	3.2	
Working	9,153	93.7		612	6.3	
**Wealth Index status**						<0.001
Poor	4,929	98.3		87	1.7	
Middle	2,756	96.0		114	4.0	
Rich	6,716	92.0		588	8.0	
**Health insurance**						<0.001
No	13,664	95.5		638	4.5	
Yes	736	83.0		151	17.0	
**Household has radio**						<0.001
No	7,431	96.1		303	3.9	
Yes	6,533	93.4		465	6.6	
**Household has television**						<0.001
No	9,429	96.4		354	3.6	
Yes	4,535	91.6		415	8.4	
**Visited healthcare facility last 12 months**						<0.001
No	6,875	96.4		260	3.6	
Yes	7,526	93.4		529	6.6	
**Pregnancy status**						0.285
No or unsure	13,275	94.7		738	5.3	
Yes	1,126	95.7		51	4.3	
**Number of living children**						<0.001
None	3,855	97.6		95	2.4	
1–4	7,900	93.4		557	6.6	
>4	2,646	95.1		137	4.9	
**Breastfeeding status**						0.122
No	11,078	94.6		635	5.4	
Yes	3,323	95.6		154	4.4	
**Zones**						<0.001
Western zone	1,246	98.4		21	1.6	
Northern zone	1,616	93.3		115	6.7	
Central zone	1,503	95.8		65	4.2	
Southern highlands	863	93.4		61	6.6	
Southern zone	773	96.3		30	3.7	
south west highlands	1,253	94.8		69	5.2	
Lake zone	4,197	95.3		209	4.7	
Eastern zone	2,458	92.7		193	7.3	
Zanzibar	490	95.0		26	5.0	

### Factors associated with breast cancer screening

Logistic regression analysis was carried out to determine the association between independent variables and breast cancer screening among the study participants. Age group(years), education, wealth index status, health insurance, residence, pregnancy status, marital status, employment status, household has radio, household has television, visited healthcare facility last 12 months, breastfeeding status, zones, number of living children were found to be significantly associated with the breast cancer screening on univariate binary logistic regression while age, wealth index, residence, education, health insurance, healthcare facility visits in the past 12 months and some of geographical zones such as northern zone, southern highlands, southwest highlands, lake zone and eastern zone on multivariate logistic regression.

Table 3 highlights the factors independently associated with breast cancer screening among women of reproductive age. In a multivariate logistic regression, older women aged 35 years and above were 5 times more likely to be screened for breast cancer (AOR = 5.33 95% CI 3.72, 7.63) compared to the younger participants aged 15–24. Participants with health insurance had 2 times higher odds of being screened for breast cancer compared to those without (AOR = 2.40, 95% CI 1.89, 3.06). Women from rich households had 2 times higher odds of breast cancer screening compared to those from poor (AOR = 2.34, 95% CI 1.61, 3.38). Women residing in rural areas were 41% less likely to be screened for breast cancer compared to those in urban areas (AOR = 0.59, 95% CI 0.46, 0.763).

**Table 3:** Factors associated with breast cancer screening among women of reproductive age in Tanzania (N = 15, 189).

Variable	Crude OR (95% CI)	Adjusted OR (95% CI)
**Age group(years)**		
15–24	ref	
25–34	3.04 (2.23, 4.14)	2.34(1.62, 3.38)
35–49	5.59(4.31, 7.25)	5.33(3.72, 7.63)
**Education**		
No education	ref	
Primary	2.63(1.85,3.74)	1.74(1.19, 2.54)
Secondary/higher	3.58(2.48, 5.18)	2.43(1.60, 3.68)
**Marital status**		
Never married	ref	
Married/living with partner	2.12(1.66, 2.72)	1.18 (0.82, 1.70)
Widowed/divorced/separated	2.91(2.08, 4.06)	1.39(0.88, 2.19)
**Residence**		
Urban	ref	
Rural	0.35(0.28,0.42)	0.59(0.46, 0.763)
**Employment status**		
Not working	ref	
Working	1.99(1.55, 2.56)	1.18(0.92, 1.53)
**Wealth Index status**		
Poor	ref	
Middle	2.36 (1.70, 3.29)	1.81(1.29, 2.56)
Rich	4.98(3.80, 6.53)	2.34(1.61, 3.38)
**Health insurance**		
No	ref	
Yes	4.39 (3.46,5.57)	2.40(1.89, 3.06)
**Household has radio**		
No	ref	
Yes	1.74(1.45, 2.09)	1.02(0.83, 1.26)
**Household has television**		
No	ref	
Yes	2.44(1.99, 2.98)	1.01(0.78, 1.32)
**Visited healthcare facility last 12 months**		
No	ref	
Yes	1.86(1.53, 2.26)	1.43(1.14, 1.78)
**Pregnancy status**		
No or unsure	ref	
Yes	0.82(0.56, 1.18)	1.11(0.75, 1.64)
**Number of living children**		
None	ref	
1–4	2.87(2.20, 3.74)	1.15(0.80, 1.64)
>4	2.11(1.54, 2.89)	0.91(0.591, 1.40)
**Breastfeeding status**		
No	ref	
Yes	0.81(0.61, 1.06)	1.09(0.79, 1.48)
**Zones**		
Western zone	ref	
Northern zone	4.28(2.37, 7.74)	2.43(1.42, 4.15)
Central zone	2.60(1.28, 5.29)	1.77(0.98, 3.17)
Southern highlands	4.23(2.32, 7.69)	2.22(1.26, 3.9)
Southern zone	2.31(1.15, 4.65)	1.70(0.89, 3.23)
Southwest highlands	3.29(1.79, 6.06)	2.18(1.27, 3.74)
Lake zone	2.99(1.68, 5.32)	2.09(1.23, 3.54)
Eastern zone	4.70(2.64, 8.37)	1.88(1.10, 3.22)
Zanzibar	3.14(1.76, 5.62)	1.57(0.91, 2.73)

## Discussion

This analytical cross-sectional study assessed factors associated with breast cancer screening among women of reproductive age in Tanzania, after controlling for other determinants the following factors remained independent significant predictors for breast cancer screening; age, wealth index, residence, education, health insurance, healthcare facility visits in the past 12 months and some of geographical zones such as northern zone, southern highlands, southwest highlands, lake zone and eastern zone.

The occurrence and fatality rates of breast cancer have experienced a rapid rise in developing nations like Tanzania and a decline in developed countries [2].Radiologist, Oncologist, breast surgeons and pathologists who play a crucial role in early detection and planning of the treatment are still scarce in Tanzania [6].

Age emerged as a very influential predictor, this could be explained by the fact that older women are vulnerable and are more knowledgeable about breast cancer screening, additionally they may also have a better understanding of the importance of preventive healthcare due to more years of exposure to health education [9, 14].This findings are consistent with previous studies which also found older women were more likely to screen for breast cancer than younger women [5]. Most of the health care facilities recommend women aged 40 years and above to undergo breast cancer screening to prevent them from breast cancer [31]. On the other hand, most SSA countries do not have national screening programs as well as enough funds to screen all eligible women [31].

In Tanzania particularly, there is unclear and unstandardized protocol for early breast cancer screening on top of that inefficient referral system adds cost and delay most of the clients with breast lesions [11]. Due to poor financial and human resources in SSA, yearly clinical breast examination to women under 40 years may be useful way to find early signs of breast cancer [32]. Previous researches have shown that 20% of breast cancer occur among women aged 30–40 years [33]. Moreover, a study indicated that in SSA only 2.2% of women aged 40–69 years had been screened for breast cancer in the past 5 years [34]. Furthermore, a study done in Sudan depicted that engaging local community volunteers for clinical breast examination might potentially enhance the early diagnosis of breast cancer in women who do not exhibit any clinical symptom [35].

As one might anticipate, and in line with previous studies [5], there was a positive association between health insurance coverage and breast cancer screening. This may be the case due to the fact that health insurance typically covers the cost of preventive services, including mammograms, reducing the out-of-pocket expenses that can deter women from seeking these screenings. Additionally, the peace of mind that comes with being insured can alleviate some of the stress and fear associated with potential medical costs, encouraging more proactive health behaviors, including regular screenings.The study found that wealth was positively associated with breast cancer screening, wealthy women are better able to afford health insurance which result in receiving preventive care services with no or minimum cost, on the other hand poor women are less likely to prioritize preventive care services over their daily needs [5]. Being rich often correlate with better access to healthcare services, including preventive measures like mammograms. Wealthier women are more likely to have health insurance, reducing financial barriers to screening. They also tend to have greater health literacy, enabling them to understand the importance of regular breast cancer screenings and to navigate healthcare systems effectively. Furthermore, higher socio-economic status is often associated with increased awareness and education about health issues, fostering a proactive approach to preventive care.

The negative association between breast cancer screening and living in the rural was not unexpected and can be explained by uneven access to healthcare services and insufficiency of health care facilities that can offer breast cancer screening compared to urban [5, 24]. Explanation for this could be the fact that accessibility to healthcare services is a significant barrier, as rural areas often have fewer medical facilities and specialized providers, requiring residents to travel long distances for screenings. This lack of local healthcare infrastructure leads to reduced opportunities for regular check-ups and preventive care, including breast cancer screening. Furthermore, social and cultural factors, such as prevailing attitudes towards medical interventions and a potential preference for alternative or home remedies, can also influence the lower screening in rural populations.

Educational attainment demonstrated a positive association with breast cancer screening, educated women may know the harmful effect of breast cancer and early detection measures [36] and this showcase the role of education in promoting health-seeking behaviors. Additionally, educated women are more likely to be informed about the risks of breast cancer, the benefits of early detection, and the availability of screening services. This knowledge empowers them to seek out and adhere to screening recommendations proactively. Furthermore, higher education often correlates with better socioeconomic status, which can provide greater access to healthcare resources, insurance coverage, and the ability to take time off work for medical appointments. The study is in accordance with earlier studies which found that educated women were more likely to be screened for breast cancer than those with no education [25].

Notably, healthcare facility visits in the past year was positively associated with breast cancer screening, this emphasizes the impact of regular health check-ups and it aligns with the findings from previous study which found that women who visited healthcare facility in the past 12 months were more likely to screen for breast cancer compared to those who didn’t [5]. Possible explanation could be, due to increased access to healthcare services and direct physician recommendations. Regular visits to healthcare facilities provide more opportunities for healthcare providers to educate women about the importance of breast cancer screening, such as mammograms, and to offer these screenings as part of routine preventive care. Furthermore, women who actively engage with healthcare services are generally more health-conscious and proactive about their health, making them more likely to follow through with recommended screenings.

### Strength and limitation

The data used for the study is a large dataset with adequate power and provides reliable nationally representative estimates that are generalizable to whole country and can be used by other African countries, the findings may contribute to improving breast cancer screening uptake among women of reproductive age. The limitation of this study include, the study was limited to only women of reproductive age 15–49 and evidence suggest that median age of breast cancer diagnosis is 62 [37].Also, the study is prone to recall bias because the response was self-reported, additionally DHS does not capture timing of the breast cancer screening which is essential for early diagnosis and treatment. Lastly, the study is a cross- sectional nature of the survey which does not allow for the determination of temporal relationships.

### Conclusion and recommendation

Very few women of reproductive age in Tanzania get screened for breast cancer, it is crucial to address this escalating burden of breast cancer through heightened health awareness, effective prevention strategies, and enhanced access to medical treatment. Older age, being wealthy, residing in rural areas, being educated, having health insurance, healthcare facility visits in the past 12 months and some of geographical zones such as northern zone, southern highlands, south west highlands, lake zone and eastern zone were independently associated with breast cancer screening. The study’s findings have significant implications for public health strategies aimed at increasing breast cancer screening. These findings suggest that focused interventions are needed to lower the disease’s incidence and increase survivor rates. It is recommended that the Tanzanian government, in collaboration with healthcare providers and non-governmental organizations, upgrade diagnostic services for breast cancer. Also, comprehensive health education programs and awareness campaigns should be implemented nationwide to improve the utilization of screening services and reduce the overall burden of breast cancer.

## Supporting information

S1 File Breast cancer screening dataset. (XLSX)

## Abbreviations

AOR: Adjusted odds ratio
CI: Confidence Interval
COR: Crude odds ratio
DHS: Demographic Health Survey

## References

[1] Lei S, Zheng R, Zhang S, Wang S, Chen R, Sun K, et al. Global patterns of breast cancer incidence and mortality: A population-based cancer registry data analysis from 2000 to 2020. Cancer Communications. 2021;41: 1183–1194. doi: 10.1002/cac2.12207 34399040 PMC8626596

[2] Sung H, Ferlay J, Siegel RL, Laversanne M, Soerjomataram I, Jemal A, et al. Global Cancer Statistics 2020 : GLOBOCAN Estimates of Incidence and Mortality Worldwide for 36 Cancers in 185 Countries. 2021;71: 209–249. doi: 10.3322/caac.21660 33538338

[3] Soerjomataram I, Bray F. Planning for tomorrow: global cancer incidence and the role of prevention 2020–2070. Nature Reviews Clinical Oncology. 2020. doi: 10.1038/s41571-021-00514-z 34079102

[4] Ginsburg O, Rositch AF, Conteh L, Mutebi M, Paskett ED, Subramanian S. Breast Cancer Disparities Among Women in Low- and Middle-Income Countries. 2020.

[5] Ba DM, Ssentongo P, Agbese E, Yang Y, Cisse R, Diakite B, et al. Prevalence and determinants of breast Saharan cancer screening in four sub- - African countries : a population- - based study. 2020; 1–8. doi: 10.1136/bmjopen-2020-039464 33046473 PMC7552834

[6] Breast Cancer Initiative. Tanzania Breast Health Care Assessment 2017: An assessment of breast cancer early detection, diagnosis and treatment in Tanzania. Available https://ww5.komen.org/breastcancertanzania. 2017; 1–62. Available: https://ww5.komen.org/uploadedFiles/_Komen/Content/Grants_Central/International_Grants/Grantee_Resources/Full_Tanzania_Assessment_report.pdf

[7] Mbonde MP, Amir H, Mbembati NA, Holland R, Schwartz-Albiez R, Kitinya JN. Characterisation of benign lesions and carcinomas of the female breast in a sub-Saharan African population. Pathology Research and Practice. 1998;194: 623–629. doi: 10.1016/s0344-0338(98)80097-6 9793961

[8] Mwakigonja AR, Rabiel H, Mbembati NA, Lema LEK. The pattern of prognostic and risk indicators among women with breast cancer undergoing modified radical mastectomy in Dar es Salaam, Tanzania. Infectious Agents and Cancer. 2016;11: 1–10. doi: 10.1186/s13027-016-0075-8 27366204 PMC4928319

[9] Morse EP, Maegga B, Joseph G, Miesfeldt S. Breast Cancer : Basic and Clinical Research. 2014; 73–79. doi: 10.4137/BCBCR.S13745.Received PMC4022700 24855371

[10] International Council for Building. Tanzania, United Republic of (TZA). International Directory of Building Research Information and Development Organizations. 2020; 220–220. doi: 10.4324/9780203974032-55

[11] Torre LA, Bray F, Siegel RL, Ferlay J, Lortet‐Tieulent J, Jemal A. Global cancer statistics, 2012. CA: A Cancer Journal for Clinicians. 2015;65: 87–108. doi: 10.3322/caac.21262 25651787

[12] MoH. National Guidelines for Early Diagnosis of Breast Cancer and Referral for Treatment. 2018.

[13] Demographic and Health Surveys. Demographic and Health Survey and Malaria Indicator Survey (TDHS-MIS). 2022.

[14] Chao CA, Huang L, Visvanathan K, Mwakatobe K, Masalu N, Rositch AF. Understanding women’s perspectives on breast cancer is essential for cancer control: Knowledge, risk awareness, and care-seeking in Mwanza, Tanzania. BMC Public Health. 2020;20: 1–11. doi: 10.1186/s12889-020-09010-y 32539723 PMC7296642

[15] Cumber SN, Nchanji KN, Tsoka-Gwegweni JM. Breast cancer among women in sub-Saharan Africa: prevalence and a situational analysis. Southern African Journal of Gynaecological Oncology. 2017;9: 35–37. doi: 10.1080/20742835.2017.1391467

[16] Black E, Richmond R. Improving early detection of breast cancer in sub-Saharan Africa: Why mammography may not be the way forward. Globalization and Health. 2019;15: 1–11. doi: 10.1186/s12992-018-0446-6 30621753 PMC6325810

[17] Abeje S, Seme A, Tibelt A. Factors associated with breast cancer screening awareness and practices of women in Addis Ababa, Ethiopia. BMC Women’s Health. 2019;19: 1–8. doi: 10.1186/s12905-018-0695-9 30616640 PMC6323829

[18] Ilo CI, Nnenna L, Nwimo IO, Onwunaka C. Breast Cancer Knowledge among Women in Ebonyi State, Nigeria : Implication for Women Breast Cancer Education Health Education Research & Development. 2020;3: 1–7. doi: 10.4172/2380-5439.1000129

[19] Viens Laura, Perin Doug, Senkomago Virginia, Neri Antonio and MS. Questions About Cervical and Breast Cancer Screening Knowledge, Practice, and Outcomes: A Review of Demographic and Health Surveys. 2018;26: 403–412. doi: 10.1089/jwh.2017.6441.Questions PMC5530254 28513340

[20] Ministry of Health. Tanzania Ministry of Health. 2023 [cited 16 Dec 2023]. Available: https://www.moh.go.tz/

[21] NBS Tanzania. National Bureau of Statistics. In: Manufacturing Index [Internet]. 2023 [cited 16 Dec 2023] pp. 4–7. Available: https://www.nbs.go.tz/index.php/en/%0A http://www.nigerianstat.gov.ng/

[22] Nyamhanga T, Eustace RW, Makoye JP, Mutalemwa K. Multi-level barriers to early detection of breast cancer among rural midlife women in Tanzania: A qualitative case study. PLoS ONE. 2024;19: 1–15. doi: 10.1371/journal.pone.0297798 38422068 PMC10903879

[23] Ministry of Health Zanzibar. Ministry of Health Zanzibar. 2023 [cited 16 Dec 2023] pp. 1–184. Available: http://coastalforests.tfcg.org/pubs/Jozani biodiversity inventory report 2002.pdf

[24] Macro International Inc. The DHS Program—DHS Questionnaires. In: Demographic and Health Surveys [Internet]. 2023 [cited 16 Dec 2023]. Available: https://dhsprogram.com/Methodology/Survey-Types/DHS-Questionnaires.cfm#CP_JUMP_16179

[25] Antabe R, Kansanga M, Sano Y, Kyeremeh E, Galaa Y. Utilization of breast cancer screening in Kenya : what are the determinants ? 2020;2: 1–9. 10.1186/s12913-020-5073-2 PMC7079358 32183801

[26] Lunani Laura L., Abaasa Andrew and GO-M. PREVALENCE AND FACTORS ASSOCIATED WITH CONTRACEPTIVE USE AMONG KENYAN WOMEN AGED 15–49 YEARS. HHS Public Access. 2019;22: 125–130. doi: 10.1007/s10461-018-2203-5.PREVALENCE PMC6132050 29943124

[27] Ogbo FA, Ogeleka P, Awosemo AO. Trends and determinants of complementary feeding practices in. 2018; 1–13. 10.1186/s41182-018-0121-x PMC6247732 30479557

[28] Ogbo FA, Ezeh OK, Awosemo AO, Ifegwu IK, Tan L, Jessa E, et al. Determinants of trends in neonatal, post-neonatal, infant, child and under-five mortalities in Tanzania from 2004 to 2016. BMC Public Health. 2019;19: 1–12. doi: 10.1186/s12889-019-7547-x 31500599 PMC6734430

[29] Mishra V, Vaessen M, Boerma JT, Arnold F, Way A, Barrere B, et al. HIV testing in national population-based surveys : experience from the Demographic and Health Surveys. 2006;029520: 537–545. doi: 10.2471/blt.05.029520 16878227 PMC2627390

[30] Macro International Inc. Measure DHS. The DHS Program—Protecting the Privacy of DHS Survey Respondents. In: DHS [Internet]. 2021 [cited 16 Dec 2023]. Available: https://dhsprogram.com/methodology/Protecting-the-Privacy-of-DHS-Survey-Respondents.cfm

[31] Lipschitz S. Screening mammography with special reference to guidelines in South Africa. 2018; 1–7. doi: 10.4102/sajr.v22i2.1370 31754518 PMC6837783

[32] More P, All M, Cancer B, Module A. Screening by Clinical Breast Examination in Western Kenya : Who Comes ? 2016;2. 10.1200/JGO.2015.000687 PMC5495450 28717690

[33] Curado M. P., B., Shin H.R, Storm H., Ferlay J.and B. Cancer Incidence in Five Continents. International Associatin of Cancer Registries. 2007;IX.

[34] Akinyemiju TF. Socio-Economic and Health Access Determinants of Breast and Cervical Cancer Screening in Low-Income Countries : Analysis of the World Health Survey. 2012;7: 3–10. doi: 10.1371/journal.pone.0048834 23155413 PMC3498259

[35] Abuidris DO, Elsheikh A, Ali M, Musa H, Elgaili E, Ahmed AO, et al. Breast-cancer screening with trained volunteers in a rural area of Sudan : a pilot study. Lancet Oncology. 14: 363–370. doi: 10.1016/S1470-2045(12)70583-1 23375833

[36] Strother RM, Asirwa FC, Busakhala NB, Njiru E, Orang E, Njuguna F, et al. The evolution of comprehensive cancer care in Western Kenya ଝ. Journal of Cancer Policy. 2013;1: e25–e30. doi: 10.1016/j.jcpo.2013.04.001

[37] Institute NC. Risk Factors: Age—NCI. 2021 [cited 20 Dec 2023]. Available: https://www.cancer.gov/about-cancer/causes-prevention/risk/age

